# Biomaterials-Based Modulation of the Immune System

**DOI:** 10.1155/2013/732182

**Published:** 2013-09-22

**Authors:** Austin B. Gardner, Simon K. C. Lee, Elliot C. Woods, Abhinav P. Acharya

**Affiliations:** Department of Bioengineering, University of California, Berkeley, CA 94720, USA

## Abstract

The immune system is traditionally considered from the perspective of defending against bacterial or viral infections. However, foreign materials like implants can also illicit immune responses. These immune responses are mediated by a large number of molecular signals, including cytokines, antibodies and reactive radical species, and cell types, including macrophages, neutrophils, natural killer cells, T-cells, B-cells, and dendritic cells. Most often, these molecular signals lead to the generation of fibrous encapsulation of the biomaterials, thereby shielding the body from these biomaterials. In this review we will focus on two different types of biomaterials: those that actively modulate the immune response, as seen in antigen delivery vehicles for vaccines, and those that illicit relatively small immune response, which are important for implantable materials. The first serves to actively influence the immune response by co-opting certain immune pathways, while the second tries to mimic the properties of the host in an attempt to remain undetected by the immune system. As these are two very different end points, each type of biomaterial has been studied and developed separately and in recent years, many advances have been made in each respective area, which will be highlighted in this review.

## 1. Immune Evasion

**Figure 1:**
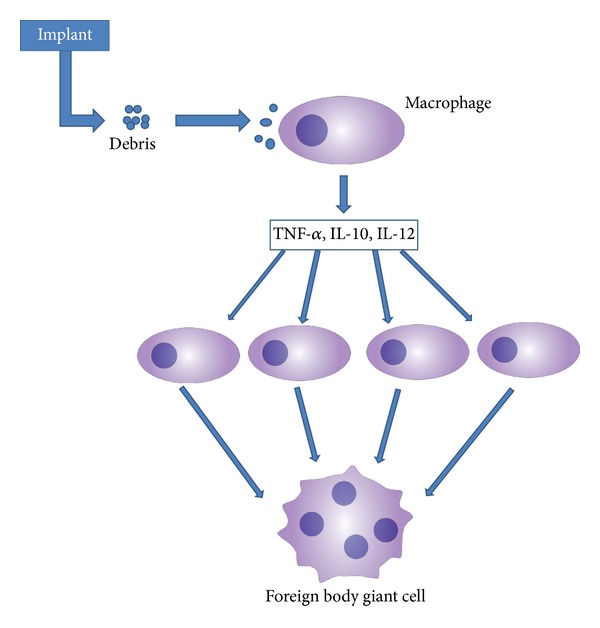
Debris generated by the wear of the implants leads to the secretion of cytokines by immune cells such as neutrophils and macrophages. These cytokines then recruit more immune cells to the site of inflammation and form foreign body giant cells. This cascading effect of inflammation leads to failure of an implant.

Since the development of the first implantable biomaterials, significant advances have been made in the field. Materials used for this purpose include metals, ceramics, and plastics, which are used in both permanent and shorter-lived biodegradable forms. Their application is wide, including metal stents implanted in arteries [1], ceramic coatings for bone tissue regeneration [2], and biodegradable polymer sutures [3]. In an ideal situation, the permanent applications do not degrade and remain as permanent implants within the body, causing no outward pathology, whereas the short-lived materials degrade into innocuous, nontoxic byproducts in a controlled manner. However, this is often not the case as these materials are foreign to the body and induce an inflammatory response. Despite the variation between the final applications, the initial response is the same. When a material is implanted into the body, a cascade of events take place in the surrounding tissue which eventually ends with the production of foreign body giant cells [4]. Immediately after the implant is placed, it is covered in a layer of host proteins in the bloodstream that enable the adhesion of host molecules of the immune response to the implant. The initial injury to the tissue surrounding the implant also induces an acute inflammatory response mediated by neutrophils. The release of cytokines, along with other factors, in response to the foreign material also leads to the activation of macrophages. In the inflammatory response, histamine-mediated phagocyte upregulation occurs, and these phagocytes can adhere to the walls of the implant. Neutrophils release proteases, lysozymes, reactive radicals, and other enzymes, leading to breakdown of any biodegradable materials. If the material is removed or degraded completely during the acute stage of inflammation, inflammation ends and the body returns to normal homeostasis. In the event the material is not removed, fully degraded or continues to trigger an inflammatory response, chronic inflammation follows [5]. This is characterized by the presence of monocytes and lymphocytes and the continuous activation of macrophages and neutrophils, which continue to release tissue-damaging enzymes and radicals. The mobilization of monocytes and macrophages is directed by chemokines and integrins. Integrins are a family of receptor molecules that mediate intercellular reactions. Adhesion to the implant is able to occur due to these integrins. Macrophages are able to phagocytose small particles generally under 50 nm, while phagocytosis of larger molecules requires the fusion of macrophages into foreign body giant cells, as shown in Figure 1. The action of these foreign body giant cells is directly linked to the degradation of implants through the removal of larger particles via phagocytosis.

While a chronic inflammatory response towards an implant can be damaging to the surrounding tissue, this may also result in failure of the implanted material and is ultimately determined by the interaction between the material and host. For example, surface properties, such as hydrophobicity, hydrophilicity, adhesive signal among others of an inorganic implant determine the type of cellular response that occurs in the host. If the surface is not biocompatible, then interactions between the surface and bodily fluid will produce inflammation due to the activation of macrophages and the secretion of cytokines, such as TNF-*α*. The body identifies the implant as foreign body and attempts to remove it via phagocytosis and neutrophil based degradation. Many natural and synthetic materials have been used as coatings for implants. Natural materials include alginate [6], chitosan [7], collagen [8], dextran [9], and hyaluronan [10]. While these materials are similar to other macromolecules in the body and their degradation leads to nontoxic products, they can often be immunogenic due to their derivation from natural sources. Popular synthetic coatings include poly(lactic acid) and poly(lactic coglycolic acid) (PLGA) [11], poly(ethylene glycol) (PEG) [12], and poly(vinyl alcohol) (PVA) [13]. These polymers can render an implant biocompatible due to their ability to prevent protein adsorption. As explained previously, by preventing protein adsorption, cell mediators of the immune system cannot recognize the material, rendering it immunologically inert. Other factors, including hydrophobicity/hydrophilicity, molecular weight, and charge density, are also important [14]. 

In addition to an immune response against the material itself, the use of implants often involves infective complications, which may be due to the direct introduction of bacteria on the implant or opportunistic infections that arise due to invasive procedures crossing immunological barriers. Common body implants that have been associated with complications are breast, hip, and knee implants. Capsular contracture can occur after breast augmentation, with bacterial infections and biofilm as the likely causes. Total hip arthroplasty can fail due to complications arising from aseptic instability which leads to immune response and inflammation. The response is largely due to B- and T-lymphocyte tissue infiltration in the tissue surrounding the implant. Knee implants elicit immune responses based on hypersensitivity reactions that result from the components of the implant itself. These complications can be reduced if the implant is modified to be biocompatible. For example, the surface of the implants can be modified to make them biologically inert or to actively affect the response of the immune system [15–17]. Modification of biomaterials with respect to their surface properties is able to improve biocompatibility and is a quickly developing subject of interest. The biological response of the host hinges upon surface interactions between the biomaterials and the biological system. Surface properties thus dictate compatibility of the material that is placed in the body. Many studies explore techniques involving surface modification of biomaterials, focusing on surface coating and its use in the fields of bone tissue engineering and tissue repair. These methods include thermal spray, electrophoresis, and pulsed laser deposition, among many others, using the materials described above. To demonstrate success, one study shows that electrophoresis coating methods are able to coat relatively complex shapes with precise control over coating thickness. There are also possible applications of these techniques in tissue repair in neurosurgery as well as orthopedic surgery [18–21].

## 2. Active Modification of Immune System

In the previous section, we reviewed methods to prevent immune responses towards implanted materials. However, there are instances where it is desirable to induce an immune response, as seen in vaccines. In a typical vaccine, the host is exposed to antigens from the pathogen orally or through intramuscular injection. This can be done using a pathogen that has been killed by chemical means or an attenuated pathogen that has been weakened in a way such that it is no longer pathogenic. Antigenic proteins can also be recombinantly synthesized in *E. coli* and used for vaccines. Regardless of how the pathogen is treated or how the antigen is obtained, the exposure of the host to these antigens induces an adaptive immune response without the risk of actual exposure to the harmful pathogen. Thus, if the host is later exposed to the pathogen, they are able to mount a rapid and effective immune response to prevent infection. This model of vaccination, while highly successful for some diseases, is problematic for others. 

The immune system employs tissue-resident antigen presenting cells to identify and present antigens to B and T cells for development of an adaptive immune response. It is well understood that the site of antigen delivery is often the location of strongest protection [22]. For example, intramuscular injection, or other forms of systemic delivery, provides poor mucosal immunity, while mucosal vaccination generates strong, albeit region-specific, mucosal protection. Most vaccines are delivered through intramuscular injection, resulting in strong protection in the tissue. However, many pathogenic diseases initially infect hosts via a mucosal membrane. Thus, it would be highly advantageous to deliver antigens via a mucosal route (oral, nasal, vaginal, or anal) to induce strong mucosal immunity. 

In addition to antigens, adjuvants are required to generate immunity. Adjuvants accomplish this by mimicking pathogen-associated molecular patterns (PAMPs), molecules that are detected by pattern recognition receptors (PRRs) located in both the membrane and cytosol of cells. This mechanism is an integral part of the innate immune system, but the pathways involved are also critical in developing a robust adaptive immunity response. PAMPs include single and double-stranded RNA, LPS, flagellin, unmethylated DNA, and particulates including silica and alum. Synthetic analogues exist for each of these PAMPs, and nanomaterials have been used to codeliver these adjuvants with antigen as a means of developing new materials for vaccine delivery. 

**Figure 2:**
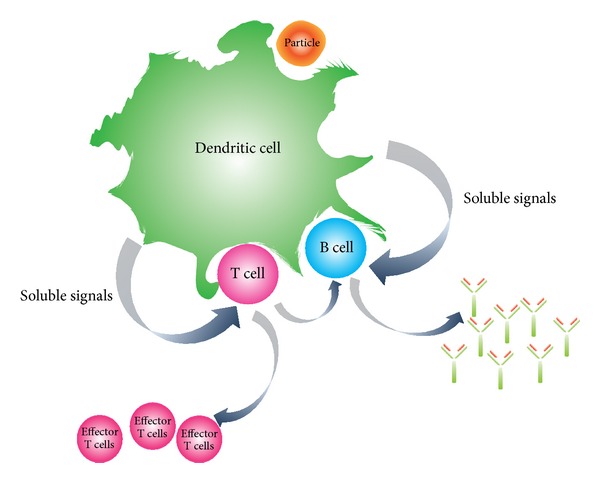
Dendritic cells can phagocytose foreign particles, process them, and induce an immune response in the form of effector T cells and B cells. This response leads to the generation of a potent vaccine.

The delivery of antigen and adjuvants to mucosal sites represents a significant challenge due to many physiological barriers. The material must cross mucus and be detected by specialized mucosa-associated tissues, composed of microfold (M) cells, which collect antigens and transfer them to nearby dendritic cells. From here, the adaptive immune response follows through to induce both cellular and humoral responses [22]. However, other barriers exist, including barriers to endocytosis by M cells and dendritic cells, and there is need for endosomal escape to the cytosol to generate a complete immune response (Figure 2). 

Materials have been developed with these considerations in mind, and various antigens and adjuvants have been delivered to a series of cellular targets. As mentioned above, the mucosal layer serves as a significant barrier to material transport to the endothelial cells below. Penetration of the layer is restricted for particles in excess of a few hundred nanometers in diameter though 50 nm particles diffuse freely across it [23]. However, particle surfaces can be modified with PEG chains to aid larger particles in penetrating the mucus. By varying the length of the chains, it was observed that shorter chains (2000 g/mol) were able to penetrate well, while longer chains (10000 g/mol) penetrated at a significantly reduced rate [24]. Furthermore, the density of the PEG grafts plays a significant role. Due to steric effects, dense layers with shorter chains have been shown to aid penetration, whereas disperse and long chains resulted in entanglement of the particle to the mucus [25].

Once past the mucus, in order to aid the delivery to dendritic cells, nanoparticles can be further modified to be specifically targeted towards dendritic cells. A newly devised strategy involves covalently linking PEG molecules to poly(lactic-co-glycolic acid) (PLGA) NPs which have also been conjugated to antibodies targeting DC-SIGN, a DC-specific marker [26]. Increasing the length of the PEG chain resulted in increased particle size and decreased the degradation rate of antigens that were encapsulated. However, PEG chains which exceed a certain length compromised the efficiency of delivery, with PEG-3000 appearing to be the most effective size.

Another key concern for the delivery of antigens and adjuvants is the wide array of cellular targets and compartments that can be targeted. For example, the PRRs which recognize nucleic acid based PAMPs are located on endosomal membranes, while others reside in the cytoplasm. Furthermore, depending on the desired MHC presentation required for the antigen, the material must either be localized to the cytoplasm (MHC class I) or the endosome/lysosome (MHC class II). Due to this, care must be taken to design materials with specific properties in order to ensure delivery to the correct cellular compartments. Luckily, the reductive environment and relative low pH of endosomes and lysosomes create opportunities for material properties to be altered or triggered in these environments. Block copolymers of PEG-SS-PPS, which contain hydrophilic and hydrophobic portions that have been joined using disulphide linkages, degrade under reductive environments, leading to release of their contents into the endosome [27]. In addition, acid-catalyzed hydrolysis of particles can be observed when using orthoester [28] and ketal [29, 30] linkages. The degradation of these materials at low pH is significantly faster than those of other polymers, and polyketals in particular have seen some success in the realm of vaccine delivery [29, 30]. In the case of MHC class I presentation, the material must reach the cytoplasm, which can be accomplished by disrupting the endosomal membrane. Cytoplasmic delivery is also important for DNA-based vaccines as antigen expression from the DNA can only occur in the cytoplasm. The methods presented previously can still apply if given enough time as PEG-SS-PPS block co-polymers will disrupt the membrane [27]. Other pH sensitive methods exist, including poly(propyl acrylic acid), which is membrane disruptive at pH 6–6.5 but not at extracellular pH, and have been used for antigen delivery towards MHC class I molecules [31]. 

Disruption can also occur through the use of polycations such as oligoarginine. Encapsulation of these materials within a pH-sensitive polyketal nanoparticle leads to release of the polycations as the nanoparticle degrades, leading to eventual cytoplasmic delivery [32]. Other polycations, such as polyethylene imine [33], can disrupt the endosome via a proton-sponge effect due to an osmotic imbalance that can occur if the material has a pKa within the range of endosomal pH. Despite the cytotoxicity of these types of polycations, techniques have been developed to use these types of materials for cytosolic delivery. By using a sequential emulsion polymerization method, core-shell nanoparticles consisting of diethylaminoethyl methacrylate cores were created and they showed cytosolic release with significantly reduced cytotoxicity [34, 35].

Adjuvant immunology combined with immunogenomic approaches are enabling progress toward rational vaccine design. However, there is a lack of an effective means to test the immune response of cells to these combinations, resulting in a hindrance in the ability to develop new vaccines. To overcome this fact, a new class of microarray composed of antigen/adjuvant-loadable PLGA microparticles is proposed as prime candidates for vaccines. The goal is to optimize particle-based vaccines designed to target DCs for disorders of the immune system [36]. 

**Figure 3:**
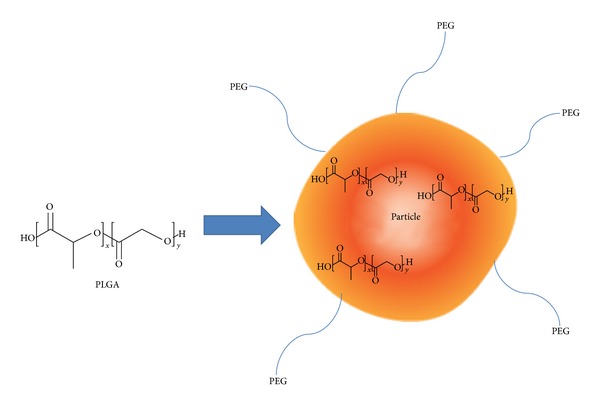
PLGA particles and scaffolds have been utilized to deliver therapeutics to the immune system. Particle-based vaccines can be generated and delivered to immune cells such as DCs to induce a potent immune response.

Developing PLGA based particles for targeted delivery and controlled release of encapsulated biological molecules is of great interest as these particles can deliver proteins or small drugs and molecules (Figure 3). The possible unique particle formulations generated by the combination of various components in a particle sharply increase as each new component is added, and there is currently no method to create libraries of unique PLGA particles. This parallel particle production methodology enables the creation of hundreds of different particle formulations with multiple coencapsulates [37]. When designing particle-based vaccines, it is important to assess probable harmful effects of the immune response on tissue at the injection site. One study used fluorescence and spectral imaging intravital microscopy of mouse window chambers to measure macrophage localization and colocalized tissue microvessel hemoglobin saturation changes in response to an immunogenic stimulus from polymer particles loaded with lipopolysaccharide (LPS) serving as a model vaccine/adjuvant system. There was faster and greater macrophage localization to inflammatory stimuli produced by LPS-loaded particle doses. This means that the immune response was taking place more quickly as compared to PLGA particles without any LPS [38].

In addition to delivering vaccines, biomaterials have been instrumental in developing immunotherapies as well. For example, it is desirable for biomaterials to generate a proinflammatory response when ex vivo DC culture is performed for cancer immunotherapy. Therefore, the surface of such culturing substrate can be modified to develop a desired response from cells. While this fact is well known, the modulation of DCs through adhesion-dependent signaling has only recently been explored. It is understood that adhesive substrates induce differential DC maturation and immune responses. For example, DCs grown on collagen and vitronectin substrates produce greater levels of IL-12p40, a proinflammatory cytokine, while DCs cultured on albumin and serum-coated tissue generate higher levels of IL-10, an anti-inflammatory cytokine. Results suggest trends of substrate dependence in DC-mediated allogeneic CD4 T(+)-cell proliferation and T-helper cell responses with modulation of IL-12p40 cytokine production. DCs play key roles in both the innate and adaptive pathways of immunity. Regulation of DC functions via cues based on adhesion has only recently been explored. It has been shown that DCs cultured on surfaces which presented integrin-targeting RGD peptide were created using “universal gradient substrate for click biofunctionalization” methodology. The findings of the study demonstrate that DCs increased production of CD86, MHC-II, IL-10, IL-12p40, and *α*V integrin binding as a function of RGD surface density, with the production of IL-12p40 being the most sensitive marker to RGD surface density [36, 39–41].

In conclusion, this review discusses the importance of biomaterials in the field of immunology. Biomaterials such as implants interact with the immune system and are a major consideration in designing immunotherapies. Biomaterials can be designed either to be “invisible” to the immune system or to actively modify it. Furthermore, biomaterials have been designed for delivering vaccines to induce a strong immune response. We believe that biomaterials will continue to play a major role in the field of immunology.

## References

[1] Weintraub WS (2007). The pathophysiology and burden of restenosis. *The American Journal of Cardiology*.

[2] Rahaman MN, Day DE, Sonny Bal B (2011). Bioactive glass in tissue engineering. *Acta Biomaterialia*.

[3] Misra SK, Valappil SP, Roy I, Boccaccini AR (2006). Polyhydroxyalkanoate (PHA)/inorganic phase composites for tissue engineering applications. *Biomacromolecules*.

[4] Brodbeck WG, Anderson JM (2009). Giant cell formation and function. *Current Opinion in Hematology*.

[5] Cobelli N, Scharf B, Crisi GM, Hardin J, Santambrogio L (2011). Mediators of the inflammatory response to joint replacement devices. *Nature Reviews Rheumatology*.

[6] de Vos P, Hoogmoed CG, Busscher HJ (2002). Chemistry and biocompatibility of alginate-PLL capsules for immunoprotection of mammalian cells. *Journal of Biomedical Materials Research*.

[7] Uchegbu IF, Schätzlein AG, Tetley L (1998). Polymeric chitosan-based vesicles for drug delivery. *The Journal of Pharmacy and Pharmacology*.

[8] Sano A, Hojo T, Maeda M, Fujioka K (1998). Protein release from collagen matrices. *Advanced Drug Delivery Reviews*.

[9] Draye JP, Delaey B, van de Voorde A, van den Bulcke A, de Reu B, Schacht E (1998). In vitro and in vivo biocompatibility of dextran dialdehyde cross-linked gelatin hydrogel films. *Biomaterials*.

[10] Vercruysse KP, Prestwich GD (1998). Hyaluronate derivatives in drug delivery. *Critical Reviews in Therapeutic Drug Carrier Systems*.

[11] Athanasiou KA, Niederauer GG, Agrawal CM (1996). Sterilization, toxicity, biocompatibility and clinical applications of polylactic acid/polyglycolic acid copolymers. *Biomaterials*.

[12] Espadas-Torre C, Meyerhoff ME (1995). Thrombogenic properties of untreated and poly(ethylene oxide)-modified polymeric matrices useful for preparing intraarterial ion-selective electrodes. *Analytical Chemistry*.

[13] Paradossi G, Cavalieri F, Chiessi E, Spagnoli C, Cowman MK (2003). Poly(vinyl alcohol) as versatile biomaterial for potential biomedical applications. *Journal of Materials Science*.

[14] Wang Y-X, Robertson JL, Spillman WB, Claus RO (2004). Effects of the chemical structure and the surface properties of polymeric biomaterials on their biocompatibility. *Pharmaceutical Research*.

[15] Lewis JS, Zaveri TD, Crooks CP, Keselowsky BG (2012). Microparticle surface modifications targeting dendritic cells for non-activating applications. *Biomaterials*.

[16] Hetrick EM, Schoenfisch MH (2006). Reducing implant-related infections: active release strategies. *Chemical Society Reviews*.

[17] Nablo BJ, Prichard HL, Butler RD, Klitzman B, Schoenfisch MH (2005). Inhibition of implant-associated infections via nitric oxide release. *Biomaterials*.

[18] Yaseen M, Zhao X, Freund A, Seifalian AM, Lu JR (2010). Surface structural conformations of fibrinogen polypeptides for improved biocompatibility. *Biomaterials*.

[19] Park JY, Gemmell CH, Davies JE (2001). Platelet interactions with titanium: modulation of platelet activity by surface topography. *Biomaterials*.

[20] Curtis A, Wilkinson C (1997). Topographical control of cells. *Biomaterials*.

[21] Wennerberg A, Albrektsson T, Johansson C, Andersson B (1996). Experimental study of turned and grit-blasted screw-shaped implants with special emphasis on effects of blasting material and surface topography. *Biomaterials*.

[22] Holmgren J, Czerkinsky C (2005). Mucosal immunity and vaccines. *Nature Medicine*.

[23] Cone RA (2009). Barrier properties of mucus. *Advanced Drug Delivery Reviews*.

[24] Lai SK, O’Hanlon DE, Harrold S (2007). Rapid transport of large polymeric nanoparticles in fresh undiluted human mucus. *Proceedings of the National Academy of Sciences of the United States of America*.

[25] Huang Y, Leobandung W, Foss A, Peppas NA (2000). Molecular aspects of muco- and bioadhesion: tethered structures and site-specific surfaces. *Journal of Controlled Release*.

[26] Cruz LJ, Tacken PJ, Fokkink R, Figdor CG (2011). The influence of PEG chain length and targeting moiety on antibody-mediated delivery of nanoparticle vaccines to human dendritic cells. *Biomaterials*.

[27] Cerritelli S, Velluto D, Hubbell JA (2007). PEG-SS-PPS: reduction-sensitive disulfide block copolymer vesicles for intracellular drug delivery. *Biomacromolecules*.

[28] Wang C, Ge Q, Ting D (2004). Molecularly engineered poly(ortho ester) microspheres for enhanced delivery of DNA vaccines. *Nature Materials*.

[29] Heffernan MJ, Kasturi SP, Yang SC, Pulendran B, Murthy N (2009). The stimulation of CD8^+^ T cells by dendritic cells pulsed with polyketal microparticles containing ion-paired protein antigen and poly(inosinic acid)-poly(cytidylic acid). *Biomaterials*.

[30] Paramonov SE, Bachelder EM, Beaudette TT (2008). Fully acid-degradable biocompatible polyacetal microparticles for drug delivery. *Bioconjugate Chemistry*.

[31] Flanary  S, Hoffman AS, Stayton PS (2009). Antigen delivery with poly(propylacrylic acid) conjugation enhances MHC-1 presentation and T-CeIl activation. *Bioconjugate Chemistry*.

[32] Cohen JL, Almutairi A, Cohen JA (2008). Enhanced cell penetration of acid-degradable particles functionalized with cell-penetrating peptides. *Bioconjugate Chemistry*.

[33] Boussif O, Lezoualc’h F, Zanta MA (1995). A versatile vector for gene and oligonucleotide transfer into cells in culture and in vivo: polyethylenimine. *Proceedings of the National Academy of Sciences of the United States of America*.

[34] Hu Y, Atukorale PU, Lu JJ (2009). Cytosolic delivery mediated via electrostatic surface binding of protein, virus, or siRNA cargos to pH-responsive core-shell gel particles. *Biomacromolecules*.

[35] Hu Y, Litwin T, Nagaraja AR (2007). Cytosolic delivery of membrane-impermeable molecules in dendritic cells using pH-responsive core-shell nanoparticles. *Nano Letters*.

[36] Acharya AP, Clare-Salzler MJ, Keselowsky BG (2009). A high-throughput microparticle microarray platform for dendritic cell-targeting vaccines. *Biomaterials*.

[37] Acharya AP, Lewis JS, Keselowsky BG (2013). Combinatorial co-encapsulation of hydrophobic molecules in poly(lactide-co-glycolide) microparticles. *Biomaterials*.

[38] Choe S-W, Acharya AP, Keselowsky BG, Sorg BS (2010). Intravital microscopy imaging of macrophage localization to immunogenic particles and co-localized tissue oxygen saturation. *Acta Biomaterialia*.

[39] Acharya AP, Dolgova NV, Clare-Salzler MJ, Keselowsky BG (2008). Adhesive substrate-modulation of adaptive immune responses. *Biomaterials*.

[40] Acharya AP, Dolgova NV, Moore NM (2010). The modulation of dendritic cell integrin binding and activation by RGD-peptide density gradient substrates. *Biomaterials*.

[41] Acharya AP, Dolgova NV, Xia CQ, Clare-Salzler MJ, Keselowsky BG (2011). Adhesive substrates modulate the activation and stimulatory capacity of non-obese diabetic mouse-derived dendritic cells. *Acta Biomaterialia*.

